# 3-Amino-1-methyl­pyrazin-1-ium iodide

**DOI:** 10.1107/S1600536809051253

**Published:** 2009-12-04

**Authors:** Daniel Foucher, Stephen Wylie, Donal H. Macartney, Alan J. Lough

**Affiliations:** a Department of Chemistry and Biology, Ryerson University, Toronto, Ontario, Canada, M5B 2K3; b Department of Chemistry, Queens University, Kingston, Ontario, Canada, K7L 3N6; c Department of Chemistry, University of Toronto, Toronto, Ontario, Canada, M5S 3H6

## Abstract

In the cation of the title compound, C_5_H_8_N_3_^+^·I^−^, the C—N(H_2_) bond distance [1.338 (8) Å] is at the lower end of the range for aryl amines. In the crystal structure, cations and anions are linked *via* N—H⋯I hydrogen bonds, forming centrosymmetric four-component clusters.

## Related literature

For the synthesis and characterization of the title compound, see: Foucher *et al.* (1993 ▶). Additional preparative details of similar compounds are given by Goto *et al.* (1968 ▶). For related structures, see Chao *et al.* (1976 ▶); Foucher *et al.* (1989 ▶); Kazheva *et al.* (2006 ▶). For the crystal structure of 3-amino-1-methylpyrazin-1-ium chloride, see the following paper. For comparative bond-distance data, see: Allen *et al.* (1987 ▶).
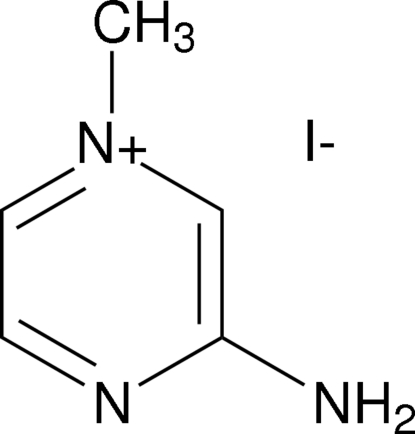


## Experimental

### 

#### Crystal data

C_5_H_8_N_3_^+^·I^−^
*M* *_r_* = 237.04
Monoclinic, 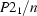

*a* = 6.9759 (5) Å
*b* = 13.2966 (15) Å
*c* = 8.3668 (9) Å
β = 90.951 (7)°
*V* = 775.96 (13) Å^3^
*Z* = 4
Mo *K*α radiation
μ = 4.05 mm^−1^
*T* = 100 K
0.20 × 0.08 × 0.06 mm

#### Data collection

Nonius KappaCCD diffractometer
Absorption correction: multi-scan (*DENZO-SMN*; Otwinowski & Minor, 1997 ▶) *T*_min_ = 0.498, *T*_max_ = 0.793
3835 measured reflections
1382 independent reflections
1020 reflections with *I* > 2σ(*I*)
*R*_int_ = 0.067

#### Refinement

*R*[*F* ^2^ > 2σ(*F* ^2^)] = 0.037
*wR*(*F* ^2^) = 0.088
*S* = 0.92
1382 reflections
92 parameters
2 restraints
H atoms treated by a mixture of independent and constrained refinement
Δρ_max_ = 1.16 e Å^−3^
Δρ_min_ = −1.33 e Å^−3^

### 

Data collection: *COLLECT* (Nonius, 2002 ▶); cell refinement: *DENZO-SMN* (Otwinowski & Minor, 1997 ▶); data reduction: *DENZO-SMN*; program(s) used to solve structure: *SIR92* (Altomare *et al.*, 1994 ▶); program(s) used to refine structure: *SHELXTL* (Sheldrick, 2008 ▶); molecular graphics: *PLATON* (Spek, 2009 ▶); software used to prepare material for publication: *SHELXTL*.

## Supplementary Material

Crystal structure: contains datablocks global, I. DOI: 10.1107/S1600536809051253/tk2585sup1.cif

Structure factors: contains datablocks I. DOI: 10.1107/S1600536809051253/tk2585Isup2.hkl

Additional supplementary materials:  crystallographic information; 3D view; checkCIF report

## Figures and Tables

**Table 1:** Hydrogen-bond geometry (Å, °)

*D*—H⋯*A*	*D*—H	H⋯*A*	*D*⋯*A*	*D*—H⋯*A*
N7—H2*N*⋯I1	0.88 (5)	2.88 (5)	3.758 (6)	173 (7)
N7—H1*N*⋯I1^i^	0.88 (5)	2.82 (5)	3.698 (6)	173 (7)

Symmetry code: (i) 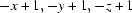.

**Fig. 1.**
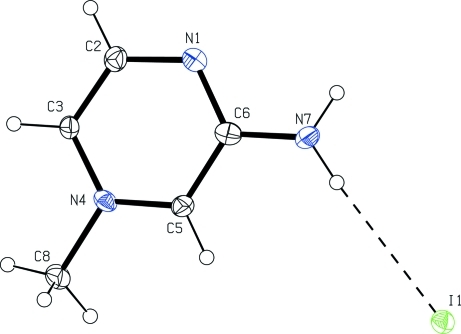
The asymmetric unit of (I) with displacement ellipsoids drawn at the 30% probability level. The dashed line indicates a hydrogen bond.

**Fig. 2.**
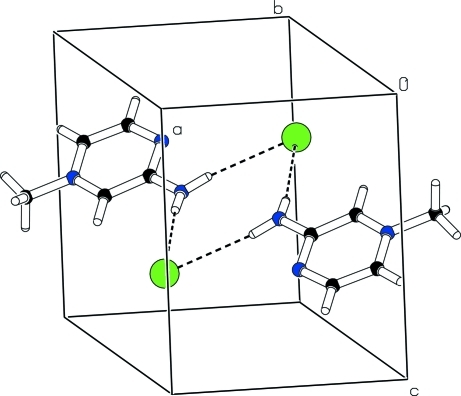
Part of the crystal structure of (I) with hydrogen bonds shown as dashed lines.

## supplementary crystallographic information

### Comment

The title compound, (I), is prepared in moderate yield from the reaction of 2-aminopyrazine with methyl iodide in carbon tetrachloride (Foucher *et al.*, 1993). The proximity of the amine group to one of the diazine nitrogen atoms makes it an ideal chelating ligand to metals and geometrically suggests the possibility for amine-imine tautomerism.

The molecular structure of (I) is shown in Fig. 1. The cation in (I) is the amine tautomer and resembles closely in terms of bond angles and bond lengths, other aromatic 1,4-diazines (Foucher *et al.*, 1989). In a comparison, the major structural difference between 2-aminopyrazine (Chao *et al.*, 1976) and (I) is observed in the C5—N4—C3 angle which is 121.3 (5)° in (I) and is 116.6 (1) in 2-aminopyrazine. These two structures are characterized by short amine-ring bond distances [1.338 (8) Å for C6—N7 in (I) and 1.341 (1) Å in 2-aminopyrazine] compared to typical bond lengths of *sp*^2^(C)—NH_2_ bond lengths, *i.e.* 1.36 Å (Allen *et al.*, 1987). These short bond lengths are suggestive of a considerable degree of double bond character, where the lone pair of the amine participates in the resonance of the ring π system. In the crystal structure, cations and anions are linked *via* intermolecular N—H···I hydrogen bonds to form centrosymmetric four component clusters (Fig. 2).

### Experimental

General procedures for the synthesis of this type of compound are given by Goto *et al.* (1968) and Kazheva *et al.* (2006). The title compound was prepared by the slow addition of an excess of methyl iodide (16 mmol) to a refluxing solution of the 2-aminopyrazine (7.9 mmol) in CCl_4_ for 12 h. The crude products were filtered off and recrystallized from a 4:1 ethanol/water mixture giving crystals suitable for X-ray analysis. Yield 1.12 g, 60%. Characterization by NMR agreed with previous literature (Foucher *et al.*, 1993).

### Refinement

H atoms bonded to C atoms were placed in calculated positions with C—H = 0.95 and 0.98 Å, and included in a riding-motion approximation with *U*_iso_(H) = 1.2*U*_eq_(C) or 1.5*U*_eq_(C_methyl_). H atoms bonded to the amine-N atom were refined independently but with the N—H distance refined as a free variable [*SHELXL* (Sheldrick, 2008) command: *DFIX* 21.00 0.01 N7 H1N N7 H2N] and with isotropic displacement parameters. The maximum and minimum residual electron density peaks of 1.15 and -1.33 eÅ^-3^, respectively are located 1.63 Å and 0.98 Å from the atoms N4 and I1, respectively.

### Crystal data

**Table:**

C_5_H_8_N_3_^+^·I^−^	*F*(000) = 448
*M**_r_* = 237.04	*D*_x_ = 2.029 Mg m^−^^3^
Monoclinic, *P*2_1_/*n*	Mo *K*α radiation, λ = 0.71073 Å
Hall symbol: -P 2yn	Cell parameters from 3835 reflections
*a* = 6.9759 (5) Å	θ = 4.1–25.4°
*b* = 13.2966 (15) Å	µ = 4.05 mm^−^^1^
*c* = 8.3668 (9) Å	*T* = 100 K
β = 90.951 (7)°	Needle, colourless
*V* = 775.96 (13) Å^3^	0.20 × 0.08 × 0.06 mm
*Z* = 4	

### Data collection

**Table:**

Nonius KappaCCD diffractometer	1382 independent reflections
Radiation source: fine-focus sealed tube	1020 reflections with *I* > 2σ(*I*)
graphite	*R*_int_ = 0.067
Detector resolution: 9 pixels mm^-1^	θ_max_ = 25.4°, θ_min_ = 4.1°
φ scans and ω scans with κ offsets	*h* = −7→7
Absorption correction: multi-scan *DENZO-SMN* (Otwinowski & Minor, 1997)	*k* = −16→16
*T*_min_ = 0.498, *T*_max_ = 0.793	*l* = −10→10
3835 measured reflections	

### Refinement

**Table:**

Refinement on *F*^2^	Primary atom site location: structure-invariant direct methods
Least-squares matrix: full	Secondary atom site location: difference Fourier map
*R*[*F*^2^ > 2σ(*F*^2^)] = 0.037	Hydrogen site location: inferred from neighbouring sites
*wR*(*F*^2^) = 0.088	H atoms treated by a mixture of independent and constrained refinement
*S* = 0.92	*w* = 1/[σ^2^(*F*_o_^2^) + (0.0489*P*)^2^] where *P* = (*F*_o_^2^ + 2*F*_c_^2^)/3
1382 reflections	(Δ/σ)_max_ < 0.001
92 parameters	Δρ_max_ = 1.16 e Å^−^^3^
2 restraints	Δρ_min_ = −1.33 e Å^−^^3^

### Special details

**Table:**

Geometry. All e.s.d.'s (except the e.s.d. in the dihedral angle between two l.s. planes) are estimated using the full covariance matrix. The cell e.s.d.'s are taken into account individually in the estimation of e.s.d.'s in distances, angles and torsion angles; correlations between e.s.d.'s in cell parameters are only used when they are defined by crystal symmetry. An approximate (isotropic) treatment of cell e.s.d.'s is used for estimating e.s.d.'s involving l.s. planes.
Refinement. Refinement of *F*^2^ against ALL reflections. The weighted *R*-factor *wR* and goodness of fit *S* are based on *F*^2^, conventional *R*-factors *R* are based on *F*, with *F* set to zero for negative *F*^2^. The threshold expression of *F*^2^ > σ(*F*^2^) is used only for calculating *R*-factors(gt) *etc*. and is not relevant to the choice of reflections for refinement. *R*-factors based on *F*^2^ are statistically about twice as large as those based on *F*, and *R*- factors based on ALL data will be even larger.

### Fractional atomic coordinates and isotropic or equivalent isotropic displacement parameters (Å^2^)

**Table:**

	*x*	*y*	*z*	*U*_iso_*/*U*_eq_	
I1	0.17537 (5)	0.57745 (3)	0.29944 (5)	0.02643 (18)	
N1	0.3523 (7)	0.1982 (4)	0.6511 (7)	0.0263 (13)	
C2	0.2488 (10)	0.1169 (5)	0.6907 (8)	0.0283 (15)	
H2A	0.3060	0.0692	0.7613	0.034*	
C3	0.0667 (9)	0.0987 (4)	0.6357 (8)	0.0234 (15)	
H3A	−0.0015	0.0405	0.6680	0.028*	
N4	−0.0138 (6)	0.1666 (4)	0.5332 (6)	0.0235 (12)	
C5	0.0814 (8)	0.2476 (5)	0.4871 (7)	0.0255 (15)	
H5A	0.0258	0.2934	0.4126	0.031*	
C6	0.2673 (8)	0.2643 (5)	0.5513 (8)	0.0266 (15)	
N7	0.3625 (8)	0.3480 (4)	0.5128 (8)	0.0309 (14)	
H1N	0.470 (8)	0.362 (6)	0.565 (8)	0.04 (2)*	
H2N	0.309 (11)	0.401 (5)	0.467 (10)	0.06 (3)*	
C8	−0.2119 (8)	0.1506 (5)	0.4716 (9)	0.0317 (17)	
H8A	−0.2840	0.2137	0.4786	0.048*	
H8B	−0.2747	0.0989	0.5355	0.048*	
H8C	−0.2078	0.1288	0.3598	0.048*	

### Atomic displacement parameters (Å^2^)

**Table:**

	*U*^11^	*U*^22^	*U*^33^	*U*^12^	*U*^13^	*U*^23^
I1	0.0231 (3)	0.0253 (3)	0.0309 (3)	0.00012 (19)	0.00021 (16)	−0.0010 (2)
N1	0.024 (3)	0.030 (3)	0.025 (3)	−0.001 (2)	0.000 (2)	−0.002 (2)
C2	0.035 (4)	0.023 (4)	0.026 (4)	−0.001 (3)	−0.002 (3)	−0.002 (3)
C3	0.023 (3)	0.018 (4)	0.030 (4)	−0.002 (2)	−0.001 (3)	−0.001 (3)
N4	0.015 (3)	0.027 (3)	0.029 (3)	0.000 (2)	0.000 (2)	−0.004 (2)
C5	0.026 (3)	0.021 (4)	0.029 (4)	0.003 (3)	−0.004 (3)	−0.001 (3)
C6	0.025 (3)	0.033 (4)	0.022 (4)	0.003 (3)	0.000 (3)	−0.003 (3)
N7	0.028 (3)	0.027 (3)	0.037 (4)	0.001 (3)	−0.003 (3)	0.011 (3)
C8	0.019 (3)	0.031 (4)	0.045 (5)	0.000 (3)	0.001 (3)	−0.001 (3)

### Geometric parameters (Å, °)

**Table:**

N1—C6	1.344 (8)	C5—C6	1.413 (8)
N1—C2	1.344 (8)	C5—H5A	0.9500
C2—C3	1.366 (9)	C6—N7	1.338 (8)
C2—H2A	0.9500	N7—H1N	0.88 (5)
C3—N4	1.360 (8)	N7—H2N	0.88 (5)
C3—H3A	0.9500	C8—H8A	0.9800
N4—C5	1.326 (8)	C8—H8B	0.9800
N4—C8	1.482 (7)	C8—H8C	0.9800
			
C6—N1—C2	116.5 (6)	N7—C6—N1	118.5 (6)
N1—C2—C3	124.1 (6)	N7—C6—C5	119.7 (6)
N1—C2—H2A	118.0	N1—C6—C5	121.8 (6)
C3—C2—H2A	118.0	C6—N7—H1N	119 (5)
N4—C3—C2	117.8 (6)	C6—N7—H2N	124 (6)
N4—C3—H3A	121.1	H1N—N7—H2N	113 (7)
C2—C3—H3A	121.1	N4—C8—H8A	109.5
C5—N4—C3	121.3 (5)	N4—C8—H8B	109.5
C5—N4—C8	118.9 (5)	H8A—C8—H8B	109.5
C3—N4—C8	119.8 (5)	N4—C8—H8C	109.5
N4—C5—C6	118.6 (6)	H8A—C8—H8C	109.5
N4—C5—H5A	120.7	H8B—C8—H8C	109.5
C6—C5—H5A	120.7		

### Hydrogen-bond geometry (Å, °)

**Table:**

*D*—H···*A*	*D*—H	H···*A*	*D*···*A*	*D*—H···*A*
N7—H2N···I1	0.88 (5)	2.88 (5)	3.758 (6)	173 (7)
N7—H1N···I1^i^	0.88 (5)	2.82 (5)	3.698 (6)	173 (7)

Symmetry codes: (i) −*x*+1, −*y*+1, −*z*+1.
